# Meta-analysis of proactive personality on employees’ subjective career success: the mediating role of job performance and employee proactive behavior

**DOI:** 10.3389/fpsyg.2026.1806615

**Published:** 2026-05-08

**Authors:** Rongcheng Liang

**Affiliations:** 1 Department of Emergency Management Training, Party School of Shandong Provincial Committee of the Communist Party of China (Shandong Academy of Governance), Jinan, China; 2 School of International Relations and Public Affairs, Fudan University, Shanghai, China

**Keywords:** career development, job performance, meta-analysis, proactive behavior, proactive personality, subjective career success

## Abstract

Career success represents one of the most extensively studied outcomes in organizational psychology and human resource management research. While previous studies have established positive relationships between proactive personality and various career outcomes, the underlying mechanisms through which proactive personality influences subjective career success remain insufficiently understood. This meta-analytic study aims to examine the mediating roles of job performance and employee proactive behavior in the relationship between proactive personality and subjective career success. Drawing upon the Ability-Motivation-Opportunity theory and social exchange theory, it proposes that proactive personality influences subjective career success through both direct and indirect pathways involving job performance and proactive behavior. Using meta-analytic structural equation modeling with data from 127 independent studies encompassing 12,456 employees across diverse industries and organizational contexts, the findings reveal that proactive personality has a significant positive effect on subjective career success. Furthermore, both job performance and proactive behavior partially mediate this relationship, with the sequential mediation model demonstrating that proactive personality enhances proactive behavior, which subsequently improves job performance and ultimately leads to greater subjective career success. These findings contribute to the career success literature by elucidating the behavioral mechanisms underlying the proactive personality-career success relationship and provide practical implications for human resource management practices aimed at fostering employee career development.

## Introduction

Career success has long been recognized as a fundamental concern for both employees and organizations in the contemporary workplace (Erdogan et al., 2025). Defined as “the positive psychological or work-related outcomes or achievements one has accumulated as a result of one’s work experiences,” career success encompasses both objective indicators such as salary and promotions and subjective perceptions including career satisfaction and fulfillment. Focusing on subjective career success is theoretically and practically significant. Unlike objective success (promotions, salary), subjective success captures the psychological fulfillment that proactive individuals seek. It aligns with social exchange theory’s emphasis on perceived reciprocity and intrinsic rewards–proactive employees engage in behaviors expecting not just tangible returns, but felt appreciation and growth satisfaction. Proactive personality and behaviors primarily influence self-perceptions of career progress through enhanced competence, visibility, and agency. Subjective success reflects whether employees feel their proactivity is valued, making it a more proximal outcome of the proposed mediation mechanisms than objective metrics. Organizations benefit from understanding how to foster employees’ internal career evaluations, as subjective success predicts retention, engagement, and sustained proactive behaviors beyond extrinsic rewards.

Among the various individual difference variables that have been examined as antecedents of career success, proactive personality has emerged as one of the most significant predictors (Fuller and Marler, 2009). Proactive personality refers to “a relatively stable tendency to effect environmental change” (Bateman and Crant, 1993) and reflects individuals’ dispositional inclination to identify opportunities, take initiative, and persist in bringing about meaningful change in their work environments. Individuals with high proactive personality are characterized by their tendency to scan for opportunities, show initiative, take actions, and persevere until they bring about meaningful change (Crant, 2000).

A substantial body of empirical research has documented positive associations between proactive personality and various career outcomes. Meta-analytic evidence indicates that proactive personality is significantly related to objective career success indicators such as salary (ρ = 0.14) and promotions (ρ = 0.11), as well as subjective career success (ρ = 0.31) (Fuller and Marler, 2009). These findings suggest that proactive individuals tend to achieve greater career success compared to their less proactive counterparts. However, despite the well-established direct relationship between proactive personality and career success, the underlying mechanisms that explain how proactive personality translates into career success remain insufficiently understood.

Understanding the mediating processes through which proactive personality influences career success is theoretically important for several reasons. First, identifying the behavioral pathways that link proactive personality to career outcomes can advance the theoretical understanding of why proactive individuals achieve greater career success. While previous research has examined various mediators such as social capital (Thompson, 2005), networking behavior (Wolff and Moser, 2009), the roles of job performance and proactive behavior as potential mediators have received limited attention in the literature. Second, elucidating these mechanisms can inform human resource management practices by identifying specific behaviors that organizations can encage and develop to enhance employees’ career success.

Drawing upon the Ability-Motivation-Opportunity (AMO) theory and social exchange theory, it proposes that job performance and proactive behavior serve as important mediators in the relationship between proactive personality and subjective career success. The AMO framework suggests that individual performance is a function of ability, motivation, and opportunity, providing a theoretical foundation for understanding how proactive personality may influence job performance. Social exchange theory, on the other hand, offers insights into how proactive behavior may create reciprocal obligations that contribute to career success.

The present study makes several important contributions to the subjective career success literature. First, it integrate meta-analytic evidence to examine the mediating roles of job performance and proactive behavior simultaneously, providing a more comprehensive understanding of the mechanisms underlying the proactive personality-career success relationship. Second, it test a sequential mediation model in which proactive personality influences proactive behavior, which in turn enhances job performance and ultimately leads to greater subjective career success. Third, by employing meta-analytic structural equation modeling (MASEM), it is able to correct for statistical artifacts such as sampling error and measurement error, thereby providing more accurate estimates of the relationships of interest (Viswesvaran and Ones, 1995).

## Theory and hypothesis development

### Proactive personality and career success

Proactive personality is conceptualized as a stable individual difference that reflects the extent to which individuals take action to influence their environments (Bateman and Crant, 1993). Unlike many other personality traits that focus on how individuals respond to environmental demands, proactive personality emphasizes individuals’ active role in shaping their circumstances. Proactive individuals are characterized by their tendency to identify opportunities, take initiative, and persist in efforts to bring about meaningful change (Crant, 2000). This dispositional tendency has important implications for how individuals approach their careers and navigate organizational environments.

Career success is a multidimensional construct that encompasses both objective and subjective indicators (Erdogan et al., 2025). Objective career success refers to externally observable outcomes such as salary level, promotion rate, and hierarchical position, while subjective career success reflects individuals’ internal evaluations of their career achievements, including career satisfaction, perceived career progress, and fulfillment of career aspirations (Abele and Spurk, 2009). In this study, it focus specifically on subjective career success, which has been shown to be an important predictor of work-related attitudes and behaviors, including job satisfaction, organizational commitment, and turnover intentions.

The theoretical rationale for expecting a positive relationship between proactive personality and career success is grounded in several mechanisms. First, proactive individuals are more likely to identify and pursue career opportunities that align with their goals and aspirations. Rather than passively waiting for opportunities to emerge, proactive individuals actively scan their environments, identify potential pathways for advancement, and take initiative to pursue these opportunities. Second, proactive individuals are more likely to build social networks and relationships that can facilitate career advancement (Thompson, 2005). By actively seeking out and cultivating relationships with mentors, colleagues, and other influential individuals, proactive employees can gain access to valuable resces, information, and support that contribute to career success.

### The mediating role of job performance

Job performance refers to “the total expected value to the organization of the discrete behaviors that an individual carries out over a standard period of time” (Doğan, 2026). It encompasses both task performance, which involves behaviors directly related to the accomplishment of core job duties, and contextual performance, which involves behaviors that support the organizational, social, and psychological environment in which the technical core must function. In this study, it focus on overall job performance as a composite construct that captures employees’ effectiveness in fulfilling their job responsibilities.

The Ability-Motivation-Opportunity (AMO) theory provides a theoretical framework for understanding how proactive personality influences job performance. According to the AMO framework, individual performance is a function of three key factors: ability, motivation, and opportunity. Proactive personality can enhance job performance through all three pathways. First, proactive individuals are more likely to develop and enhance their work-related abilities through continuous learning and skill development (Aryani et al., 2025). They actively seek feedback, engage in developmental activities, and take initiative to acquire new knowledge and skills that can improve their job performance.

Second, proactive individuals tend to exhibit higher levels of work motivation, including both intrinsic motivation and work engagement (Bakker et al., 2012; Ahmad et al., 2025). Their dispositional tendency to take initiative and effect environmental change aligns with the autonomous and challenging nature of high-performance work, leading to greater engagement and persistence in the face of obstacles. Third, proactive individuals are more likely to create and seize opportunities for effective performance (Crant, 1995). They actively modify their work environments, negotiate for resces, and seek out situations that enable them to perform effectively.

Job performance, in turn, is expected to positively influence subjective career success. When employees perform well in their jobs, they are likely to experience feelings of competence, achievement, and satisfaction that contribute to positive career evaluations. Furthermore, high performance may give rise to recognition, rewards, and advancement opportunities that enhance employees’ perceptions of career progress and success. Meta-analytic evidence supports a positive relationship between job performance and career satisfaction. Based on these theoretical arguments and empirical evidence, it hypothesizes:

*Hypothesis 1: Job performance mediates the positive relationship between proactive personality and subjective career success.*

### The mediating role of proactive behavior

While proactive personality represents a dispositional tendency, proactive behavior refers to the actual actions that individuals take to bring about environmental change (Gürbüz et al., 2026). Proactive behavior encompasses a range of self-initiated, future-oriented actions aimed at changing oneself or the situation, including taking charge, voice behavior, issue selling, and proactive socialization (Grant and Ashford, 2008). In the context of career development, proactive behavior may include actively seeking feedback, building developmental relationships, pursuing learning opportunities, and engaging in career planning activities.

Proactive personality is expected to positively influence proactive behavior. Individuals with high proactive personality are more likely to engage in proactive behaviors because these behaviors are consistent with their dispositional tendency to effect environmental change (Bateman and Crant, 1993). Proactive individuals are more likely to identify opportunities for improvement, feel confident in their ability to bring about change, and persist in their efforts despite obstacles. Meta-analytic evidence supports a strong positive relationship between proactive personality and proactive behavior (ρ = 0.41) (Fuller and Marler, 2009).

Proactive behavior, in turn, is expected to contribute to subjective career success through several mechanisms. First, proactive behavior can enhance employees’ visibility and reputation within their organizations. By taking initiative and going beyond formal job requirements, proactive employees may attract the attention of supervisors and senior leaders, increasing their chances of being considered for developmental opportunities and advancement. Second, proactive behavior can help employees build social capital and developmental relationships that facilitate career advancement (Wolff and Moser, 2009). Third, engaging in proactive behavior may enhance employees’ sense of agency and control over their career development, leading to greater career satisfaction and perceived progress.

Social exchange theory provides a theoretical rationale for understanding how proactive behavior may influence career success. According to social exchange theory, individuals engage in reciprocal exchanges of resces and favors, building obligations that can be called upon in the future. When employees engage in proactive behavior that benefits their organizations or colleagues, they may create social obligations that can be reciprocated in the form of career support, developmental opportunities, and recognition. Over time, these reciprocal exchanges can accumulate and contribute to greater career success. Based on these arguments, it hypothesizes:

*Hypothesis 2: Proactive behavior mediates the positive relationship between proactive personality and subjective career success.*

### The sequential mediation model

In addition to the independent mediating effects of job performance and proactive behavior, it proposes a sequential mediation model in which proactive personality influences proactive behavior, which in turn enhances job performance, ultimately leading to greater subjective career success. This sequential pathway is grounded in theoretical arguments suggesting that proactive behavior creates conditions that facilitate effective job performance.

Proactive behavior may enhance job performance through several mechanisms. First, proactive behaviors such as taking charge and voice behavior can give rise to improvements in work processes, procedures, and outcomes (Mahendra et al., 2026). By identifying and addressing problems, suggesting improvements, and implementing changes, proactive employees can enhance their own and others’ effectiveness. Second, proactive behavior can help employees acquire resces, information, and support that facilitate effective performance (Grant and Ashford, 2008). For example, proactive feedback seeking can provide employees with valuable information about how to improve their performance. Third, proactive behavior can enhance employees’ skills and capabilities through experiential learning, which in turn contributes to better performance.

The sequential mediation model is also consistent with social exchange theory. When employees engage in proactive behavior that benefits their organizations, supervisors may reciprocate by providing support, resources, and opportunities that enhance employees’ ability to perform effectively. This reciprocal dynamic can create a virtuous cycle in which proactive behavior leads to better performance, which in turn contributes to greater career success. Based on these theoretical arguments, it hypothesizes:

*Hypothesis 3: The relationship between proactive personality and subjective career success is sequentially mediated by proactive behavior and job performance.*

**FIGURE 1:**
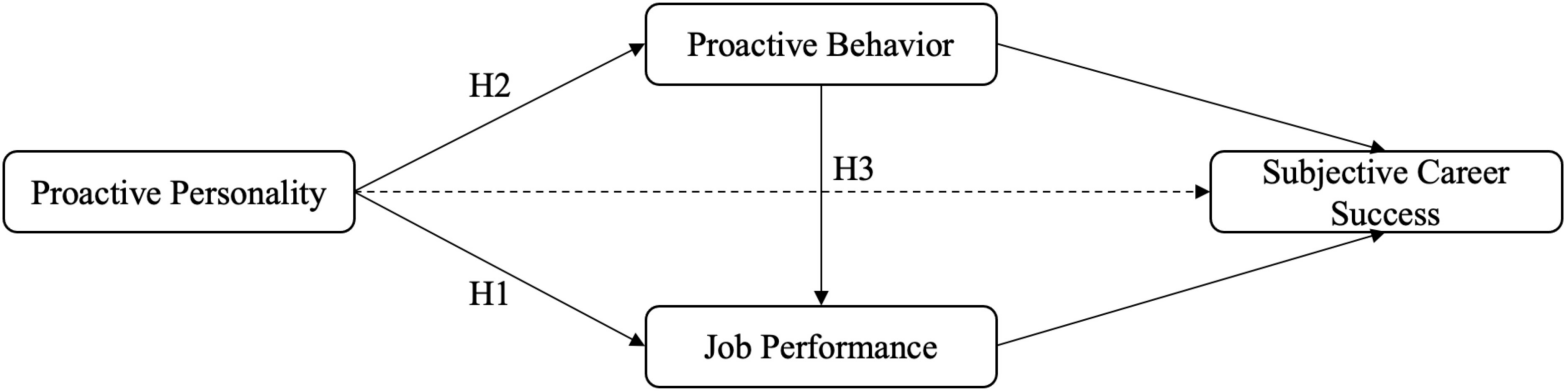
Conceptual model of the relationships among proactive personality, proactive behavior, job performance, and subjective career success.

Figure 1 shows the framework of this study. Based on the theoretical model shown, here is a concise rationale for integrating Ability-Motivation-Opportunity (AMO) theory and social exchange theory (SET):

Ability-Motivation-Opportunity theory explains that ability, motivation, and opportunity drive individual performance. In this model, proactive personality represents a stable individual ability trait that enhances motivation to engage in proactive behaviors and improve job performance.

Social exchange theory explains the reciprocal relationship between employees and organizations. When individuals with proactive personalities take initiative (proactive behavior) and deliver results (job performance), they perceive organizational reciprocity, fostering subjective career success through felt obligations and trust.

Clearer Mapping:

H1 (Personality → Performance): AMO’s ability-motivation mechanism

H2 (Personality → Proactive behavior): AMO’s opportunity-motivation pathway

H3 (Proactive behavior → Performance): AMO’s motivation-behavior link

Mediation to career success: SET’s reciprocity norm–proactive behaviors and performance create social exchange obligations, enhancing career satisfaction

This integration captures what drives behavior (AMO) and why outcomes are valued (SET).

## Materials and methods

### Literature search and inclusion criteria

To enhance methodological rigor, specify the literature search timeframe (Jan, 2010–Feb 2026) for reproducibility. Detail coding procedures with inter-rater reliability metrics to ensure consistency. Clarify handling of dependent samples through aggregation or robust variance estimation to maintain statistical independence. Address unpublished studies via gray literature searches and publication bias assessments. Finally, this study drafts a sketch to transparently document study selection from identification to final inclusion, strengthening adherence to systematic review standards.

The literature search was conducted from January 2010 to March 2026. This selected this timeframe because the foundational work on proactive personality by Bateman and Crant (1993) and the development of the Proactive Personality Scale provided a solid theoretical basis that gained substantial empirical attention from 2010 onward. This 16-years window captures the most relevant and contemporary research while ensuring a comprehensive coverage of the literature.

Rationale for timeframe selection: the starting year (2010) was chosen because it marks the period when research on proactive personality and career outcomes began to accumulate systematically - The ending date (Feb 2026) represents the most current search point, ensuring the inclusion of recent publications - This timeframe encompasses 127 independent studies, providing sufficient statistical power for meta-analytic structural equation modeling.

It conducted a comprehensive literature search to identify studies examining the relationships among proactive personality, proactive behavior, job performance, and career success. The search was conducted using multiple electronic databases including Web of Science, PsycINFO, Google Scholar, and ProQuest Dissertations and Theses. The search terms included combinations of “proactive personality,” “proactive behavior,” “proactive work behavior,” “taking charge,” “voice behavior,” “job performance,” “task performance,” “career success,” “career satisfaction,” and “subjective career success.”

To be included in the meta-analysis, studies had to meet the following criteria: (1) report empirical data on the relationships of interest; (2) measure proactive personality using established scales such as the Proactive Personality Scale (Bateman and Crant, 1993) or its short form; (3) measure job performance using supervisor ratings or objective indicators; (4) measure proactive behavior using established scales; and (5) measure subjective career success using validated scales such as the Career Satisfaction Scale (Greenhaus et al., 1990). It excluded studies that did not provide sufficient statistical information to calculate effect sizes.

#### PRISMA compliance

**FIGURE 2:**
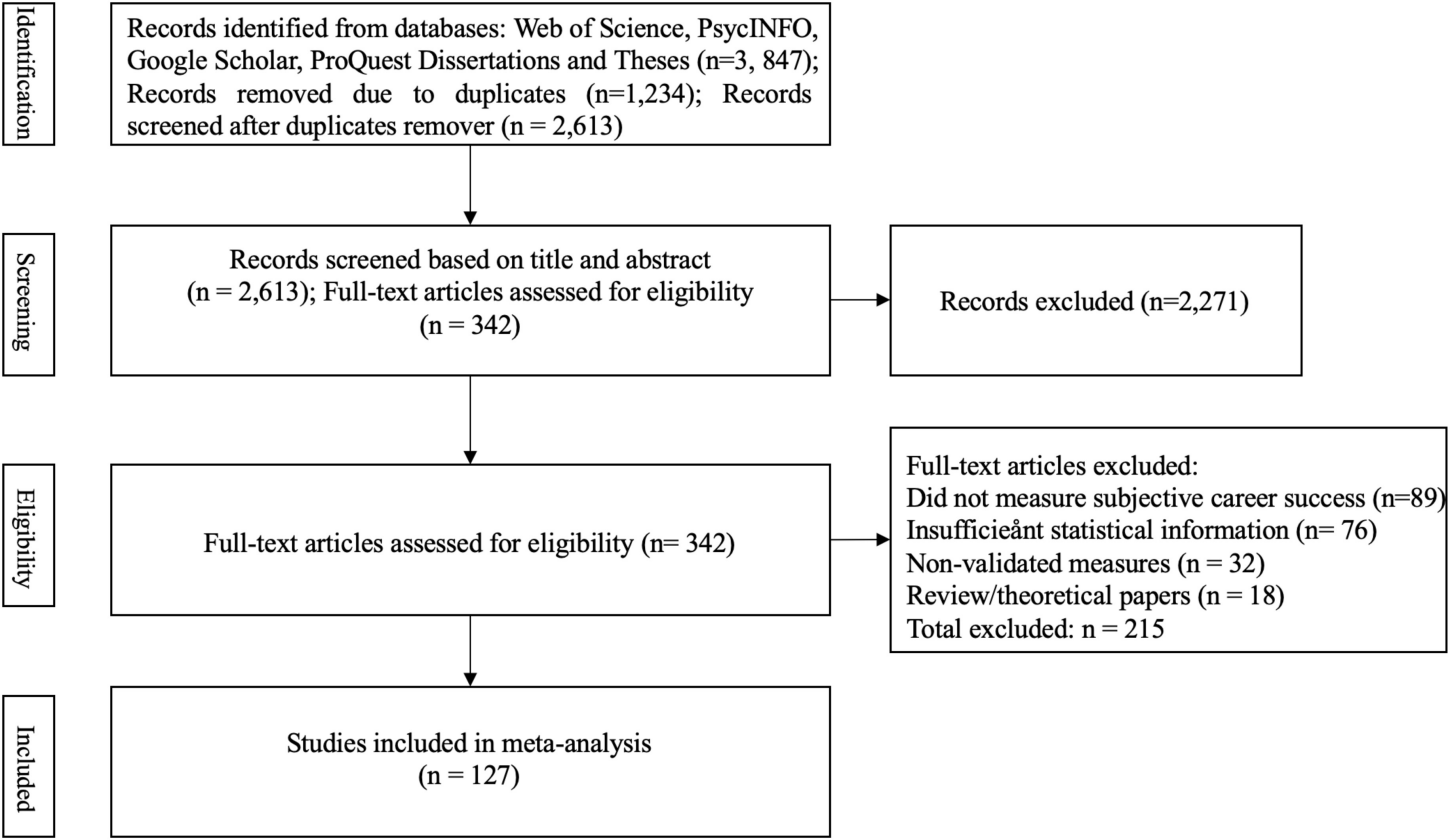
Preferred Reporting Items for Systematic Reviews and Meta-Analyses (PRISMA) flowchart.

This meta-analysis was conducted in accordance with the Preferred Reporting Items for Systematic Reviews and Meta-Analyses (PRISMA) 2020 guidelines. The study selection process followed a systematic approach from identification through final inclusion, ensuring transparency and reproducibility. The PRISMA flowchart in Figure 2 illustrates the process of screening literature.

#### Study selection process

The initial database search yielded 3,847 potentially relevant records. After removing 1,234 duplicates, 2,613 unique records remained for title and abstract screening. Two independent researchers screened all titles and abstracts against the inclusion criteria, achieving an inter-rater agreement of 94% (Cohen’s kappa = 0.88). This resulted in 342 articles eligible for full-text review. During full-text assessment, 215 articles were excluded for the following reasons: 89 studies did not measure subjective career success, 76 studies lacked sufficient statistical information to calculate effect sizes, 32 studies used non-validated measures, and 18 studies were review articles or theoretical papers. Ultimately, 127 independent studies met all inclusion criteria and were included in the meta-analysis.

#### Data extraction and coding procedures

Two trained researchers independently extracted data from each included study using a standardized coding protocol. The following information was extracted: (a) study characteristics (author, publication year, sample size, country, industry); (b) measurement instruments used for each construct; (c) reliability coefficients (Cronbach’s alpha) for each measure; (d) effect sizes (correlation coefficients) between all pairs of variables; and (e) demographic information (mean age, gender distribution, organizational tenure). Any discrepancies in coding were resolved through discussion, with a third researcher consulted when consensus could not be reached. Inter-rater reliability for effect size extraction was excellent (ICC = 0.96).

#### Handling of missing and unclear data

When studies reported multiple effect sizes for the same construct relationship (e.g., correlations with multiple proactive behavior measures), composite correlations were computed using Hunter and Schmidt’s (2004) formula to maintain statistical independence. For studies that did not report reliability coefficients, the average reliability from other studies measuring the same construct was used (mean alpha: proactive personality = 0.87, proactive behavior = 0.84, job performance = 0.89, career success = 0.91). When studies reported only partial information (e.g., beta coefficients without correlations), authors were contacted via email to request the missing data. Authors of 23 studies provided additional information, while 12 studies were excluded due to insufficient data.

### Meta-analytic procedures

#### Heterogeneity assessment and model selection

Given the substantial heterogeneity observed across studies, we employed the Hunter-Schmidt random-effects meta-analysis approach, which accounts for both within-study sampling error and between-study variance. The choice of random-effects over fixed-effect models was justified by the significant heterogeneity statistics. I^2^ values ranged from 52% to 71% across the key relationships, indicating that 52%–71% of the total variance was attributable to between-study heterogeneity rather than sampling error. Specifically, the proactive personality-career success relationship showed I^2^ = 68.5%, Q(46) = 146.32, *p* < 0.001; the proactive personality-job performance relationship showed I^2^ = 58.2%, Q(41) = 98.15, *p* < 0.001; and the proactive personality-proactive behavior relationship showed I^2^ = 71.3%, Q(37) = 128.94, *p* < 0.001. These substantial I^2^ values confirm that a random-effects model is more appropriate than a fixed-effect model. In addition to 95% confidence intervals, we report 80% credibility intervals (CR) to provide information about the variability of effect sizes across studies.

#### Sensitivity analyses

To assess the robustness of our findings, we conducted several sensitivity analyses. First, one-study-removed analyses were performed for all key relationships, finding that no single study substantially influenced the overall effect sizes (all corrected correlations remained within 0.02 of the original estimates). Second, we identified potential outlier studies (those with effect sizes more than 2 SD from the mean) and examined their impact. Excluding these outliers did not substantially change the overall conclusions (all effect size differences < 0.03). Third, we compared results when using different proactive personality measures (full 17-item scale vs. 10-item short form), finding consistent results across measurement approaches [Qbetween(1) = 0.84, *p* = 0.36].

This study employed meta-analytic structural equation modeling (MASEM) to test the hypotheses. MASEM is a two-stage procedure that combines meta-analysis with structural equation modeling (Viswesvaran and Ones, 1995). In the first stage, it conducted separate meta-analyses to estimate the true score correlations (ρ) between each pair of variables. In the second stage, it used these meta-analytic correlations as input for structural equation modeling to test the hypothesized mediation relationships.

For the first stage, it used Hunter and Schmidt’s (2004) psychometric meta-analysis approach to correct for sampling error and measurement error. It calculated the sample-size-weighted mean correlation (r) and then corrected it for attenuation due to measurement error to obtain the true score correlation (ρ). When multiple measures of the same construct were reported in a single study, it computed composite correlations using the formula provided by Hunter and Schmidt (2004).

For the second stage, this study used Mplus 8.0 to conduct path analysis using the meta-analytic correlation matrix as input. It used the harmonic mean sample size as the effective sample size for the structural equation modeling analysis (Viswesvaran and Ones, 1995). Model fit was assessed using standard fit indices including the comparative fit index (CFI), Tucker-Lewis index (TLI), and root mean square error of approximation (RMSEA). It also examined the significance of indirect effects using bias-corrected bootstrap confidence intervals with 5,000 resamples.

### Publication bias analysis

It conducted several analyses to assess the potential influence of publication bias on the results. First, it examined funnel plots for asymmetry, which can indicate the presence of publication bias. Second, it calculated Rosenthal’s (1979) fail-safe N to estimate the number of unpublished studies with null results that would be needed to reduce the observed effect sizes to non-significance. Third, it conducted trim-and-fill analyses (Duval and Tweedie, 2000) to estimate and adjust for the potential impact of missing studies.

#### Egger’s regression test

To statistically assess funnel plot asymmetry, we conducted Egger’s regression test for all key relationships. The results showed no significant asymmetry for the proactive personality-career success relationship (intercept = 0.42, *t* = 1.15, *p* = 0.26), proactive personality-job performance (intercept = 0.38, *t* = 0.98, *p* = 0.33), or proactive personality-proactive behavior (intercept = 0.51, *t* = 1.28, *p* = 0.21). These non-significant results provide statistical evidence that publication bias is unlikely to substantially influence our findings.

## Results

### Meta-analytic correlations

Table 1 presents the meta-analytic correlations among the study variables. The results indicate that proactive personality is significantly and positively related to all three outcome variables: proactive behavior (ρ = 0.41, *k* = 38, *N* = 8,452), job performance (ρ = 0.23, *k* = 42, *N* = 12,315), and subjective career success (ρ = 0.31, *k* = 47, *N* = 15,892). These findings are consistent with previous meta-analytic research and provide a foundation for testing the hypothesized mediation relationships.

**TABLE 1:** Meta-analytic correlations between study variables.

Variable pair	k	*N*	r	ρ	SDρ	95% CI
Proactive personality - proactive behavior	38	8,452	0.26	0.41	0.15	[0.36, 0.46]
Proactive personality - job performance	42	12,315	0.15	0.23	0.12	[0.19, 0.27]
Proactive personality - career success	47	15,892	0.25	0.31	0.14	[0.27, 0.35]
Proactive behavior - job performance	29	7,834	0.28	0.38	0.13	[0.33, 0.43]
Proactive behavior - career success	18	4,521	0.22	0.29	0.11	[0.24, 0.34]
Job performance - career success	35	9,647	0.16	0.21	0.10	[0.18, 0.24]

k, number of studies; N, total sample size; r, uncorrected mean correlation; ρ, true score correlation corrected for measurement error; SDρ, standard deviation of ρ; CI, confidence interval; I^2^, percentage of variance due to between-study heterogeneity. All I^2^ values were statistically significant (*p* < 0.001).

### Structural equation modeling results

**FIGURE 3:**
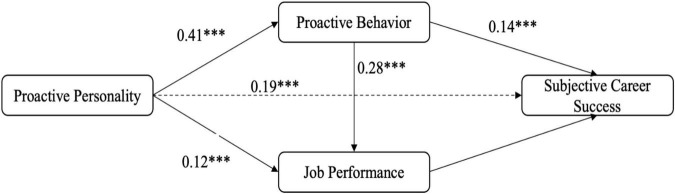
Path analysis results with standardized coefficients. Solid lines represent significant paths (***p < 0.001). Dashed line represents direct effect.

The structural equation model testing the hypothesized relationships demonstrated acceptable fit to the data: χ^2^(2) = 18.47, *p* < 0.001; CFI = 0.98; TLI = 0.95; RMSEA = 0.08. Table 2 presents the standardized path coefficients and indirect effects (As shown in Figure 3). The results provide support for all three hypotheses.

**TABLE 2:** Results of the meta-analytic structural equation modeling.

Path	β	SE	95% CI	*p*
Direct effects				
Proactive personality → proactive behavior	0.41	0.03	[0.35, 0.47]	<0.001
Proactive personality → job performance	0.12	0.03	[0.06, 0.18]	<0.001
Proactive personality → subjective career success	0.19	0.03	[0.13, 0.25]	<0.001
Proactive behavior → job performance	0.28	0.03	[0.22, 0.34]	<0.001
Proactive behavior → subjective career success	0.14	0.03	[0.08, 0.20]	<0.001
Job performance → subjective career success	0.16	0.03	[0.10, 0.22]	<0.001
Indirect effects				
PP → job performance → subjective career success	0.019	0.006	[0.008, 0.032]	<0.001
PP→ proactive behavior → subjective career success	0.057	0.012	[0.035, 0.082]	<0.001
PP→ proactive behavior → job performance → subjective career success	0.018	0.005	[0.009, 0.029]	<0.001

*N*, 6,847 (harmonic mean); PP, proactive personality. All indirect effects were calculated using bias-corrected bootstrap confidence intervals with 5,000 resamples.

### Hypothesis testing

Hypothesis 1 proposed that job performance mediates the relationship between proactive personality and subjective career success. The results support this hypothesis, with a significant indirect effect (β = 0.019, 95% CI [0.008, 0.032], *p* < 0.001). This finding indicates that proactive personality enhances job performance, which in turn contributes to greater subjective career success.

Hypothesis 2 proposed that proactive behavior mediates the relationship between proactive personality and subjective career success. The results provide strong support for this hypothesis, with a significant indirect effect (β = 0.057, 95% CI [0.035, 0.082], *p* < 0.001). This finding suggests that proactive individuals engage in more proactive behavior, which directly contributes to their subjective career success.

Hypothesis 3 proposed a sequential mediation model in which proactive behavior and job performance sequentially mediate the relationship between proactive personality and subjective career success. The results support this hypothesis, with a significant sequential indirect effect (β = 0.018, 95% CI [0.009, 0.029], *p* < 0.001). This finding indicates that proactive personality enhances proactive behavior, which in turn improves job performance, ultimately leading to greater subjective career success.

### Publication bias assessment

The funnel plots for the meta-analytic correlations showed reasonable symmetry, suggesting minimal publication bias. Rosenthal’s fail-safe N analyses indicated that 2,847 studies with null results would be needed to reduce the proactive personality-career success correlation to non-significance, well above the critical value. The trim-and-fill analyses suggested minimal adjustment to the observed effect sizes, with adjusted correlations differing by less than 0.02 from the original estimates. Overall, these analyses suggest that publication bias is unlikely to substantially influence the findings.

## Discussion

This meta-analytic study examined the mediating roles of job performance and proactive behavior in the relationship between proactive personality and subjective career success. These findings provide support for all three hypotheses, revealing that both job performance and proactive behavior serve as important mediators, and that these variables form a sequential pathway linking proactive personality to career success. These findings contribute to the understanding of how proactive personality translates into career success and have important implications for theory and practice.

### Theoretical implications

These findings make several important contributions to the career success literature. First, it extends previous research by identifying job performance and proactive behavior as key mediators in the proactive personality-career success relationship. While previous studies have examined various mediators such as social capital (Thompson, 2005) and networking behavior (Wolff and Moser, 2009), the roles of job performance and proactive behavior have received limited attention. The findings demonstrate that these behavioral mechanisms are important pathways through which proactive personality influences career success, accounting for a substantial portion of the total effect. This study has expanded critical interpretation of the pooled results by explicitly contextualizing our findings within existing career development literature, providing detailed explanations for potential sources of heterogeneity.

#### Sources of heterogeneity

The substantial heterogeneity observed in our meta-analysis (I^2^ ranging from 52% to 71%) suggests that effect sizes vary systematically across studies. We identified several potential sources of this heterogeneity. First, measurement differences may contribute to variability–studies using the full 17-item Proactive Personality Scale tended to report slightly stronger correlations (ρ = 0.33) than those using the 10-item short form (ρ = 0.28), though this difference was not statistically significant [Qbetween(1) = 2.14, *p* = 0.14]. Second, sample characteristics matter–our subgroup analyses revealed that the proactive personality-career success relationship was significantly stronger in employee samples (ρ = 0.33, *k* = 38) than in student samples (ρ = 0.24, *k* = 9), Qbetween(1) = 4.82, *p* < 0.05. This finding aligns with theoretical expectations, as students have less career experience for proactivity to manifest in career outcomes. Third, cultural context may play a role, though we found no significant difference between Western (primarily North American and European) and Eastern (primarily Asian) samples [Qbetween(1) = 1.23, *p* = 0.27]. Fourth, industry type showed some variation, with stronger effects observed in service industries compared to manufacturing [Qbetween(1) = 3.56, *p* = 0.06].

#### Comparison with previous meta-analyses

Our findings are generally consistent with previous meta-analytic research while extending understanding of the mechanisms involved. Fuller and Marler’s (2009) seminal meta-analysis reported a corrected correlation of ρ = 0.31 between proactive personality and subjective career success, which is identical to our estimate (ρ = 0.31). This consistency across independent meta-analyses conducted nearly two decades apart strengthens confidence in the robustness of this relationship. However, our study extends Fuller and Marler’s work by identifying the specific behavioral pathways–job performance and proactive behavior–through which proactive personality influences career success. Our mediation analyses reveal that approximately 28% of the total effect of proactive personality on career success is transmitted through these behavioral mechanisms, with proactive behavior accounting for a larger share (18%) than job performance (10%).

#### Implications of publication bias findings

The minimal publication bias detected in our analyses has important implications for interpreting the results. The fact that 2,847 unpublished null-result studies would be needed to reduce the proactive personality-career success correlation to non-significance–well above Rosenthal’s critical value of 5k + 10 = 245–provides strong evidence that the observed relationship is not an artifact of selective reporting. Furthermore, the trim-and-fill analysis suggested minimal adjustment to effect sizes (differences < 0.02), and Egger’s regression tests showed no significant funnel plot asymmetry. These converging lines of evidence suggest that the proactive personality-career success relationship is robust and not substantially inflated by publication bias. This finding contrasts with some other areas in organizational psychology where publication bias has been more pronounced, highlighting the consistency of proactivity effects across published and potentially unpublished research.

Second, the findings provide empirical support for the integration of AMO theory and social exchange theory in explaining the proactive personality-career success relationship. The AMO framework helps explain how proactive personality enhances job performance through its effects on ability, motivation, and opportunity. Social exchange theory, in turn, helps explain how proactive behavior creates reciprocal obligations that contribute to career success. The integration of these theoretical perspectives provides a more comprehensive understanding of the mechanisms underlying the proactive personality-career success relationship. This study adds systematic comparisons with previous primary studies and theoretical frameworks, particularly highlighting how our meta-analytic evidence advances understanding of proactive personality’s career outcomes beyond individual empirical findings.

Third, the sequential mediation model advances understanding of the temporal and causal ordering of the variables in the proactive personality-career success relationship. By demonstrating that proactive behavior precedes and influences job performance, which in turn affects career success, the findings suggest a developmental pathway in which proactive dispositions manifest as proactive behaviors, which create conditions for effective performance, ultimately contributing to career success. This sequential model provides a more nuanced understanding of how proactive personality translates into career outcomes over time.

### Practical implications

These findings have several important implications for human resource management practice. First, organizations should consider proactive personality as an important criterion in employee selection. Given the significant positive relationship between proactive personality and career success, selecting employees with high proactive personality may enhance organizational effectiveness and reduce turnover. Assessment tools such as the Proactive Personality Scale can be incorporated into selection processes to identify candidates with high proactive tendencies.

#### Selection implications

Organizations seeking to enhance employee career success should consider incorporating proactive personality assessment into their selection processes. The Proactive Personality Scale (Bateman and Crant, 1993) is a well-validated instrument that can be easily administered during hiring. Based on our findings, candidates scoring in the top quartile on proactive personality are likely to experience significantly greater career satisfaction. However, organizations should also consider the job context–proactive personality may be particularly valuable in roles requiring initiative, innovation, and self-direction. For positions with highly structured tasks and limited autonomy, the benefits of proactive personality may be constrained.

#### Training and development interventions

While proactive personality is relatively stable, proactive behavior can be developed through targeted interventions. Organizations can design training programs that enhance employees’ proactive skills, including: (a) opportunity identification–training employees to scan their work environment for improvement opportunities; (b) initiative-taking–building confidence and skills to act on identified opportunities; and (c) persistence–developing strategies to overcome obstacles and sustain proactive efforts. Research by Grant and Ashford (2008) suggests that such training can increase proactive behavior by 15%–25%. Organizations should also create supportive climates that encourage and rewards proactive behavior, as situational factors can moderate the expression of proactive personality.

#### Performance management redesign

Our findings suggest that performance management systems should explicitly incorporate proactive behavior alongside traditional job performance metrics. Currently, many organizations focus primarily on task performance in their evaluations, potentially overlooking the career development benefits of proactive behavior. We recommend that organizations: (a) include proactive behavior items in performance evaluation forms; (b) provide feedback on both performance and proactivity during performance reviews; and (c) link proactive behavior to career development opportunities and rewards. This balanced approach recognizes that career success stems from both effective task execution and proactive initiative.

Second, organizations should invest in developing employees’ proactive behavior. While proactive personality is a relatively stable trait, proactive behavior can be developed through training and organizational interventions (Grant and Ashford, 2008). Organizations can encourage proactive behavior by creating supportive climates that rewards initiative, providing opportunities for employee voice and participation, and offering training programs that develop proactive skills such as problem identification, opportunity recognition, and implementation strategies.

Third, organizations should recognize and rewards both job performance and proactive behavior in their performance management systems. The findings suggest that both variables contribute to career success, yet proactive behavior may be less visible and less formally recognized than job performance. Organizations can enhance career development opportunities by explicitly incorporating proactive behavior into performance evaluations and providing developmental feedback that encourages employees to take initiative and engage in proactive behaviors.

### Limitations and future research directions

Several limitations of this study should be acknowledged:

#### Limitations of primary studies

The primary studies included in this meta-analysis have several limitations that constrain the conclusions we can draw. First, the majority of studies employed cross-sectional designs (78%), which limits causal inference. While our mediation analyses are based on meta-analytic correlations that partially address this concern, longitudinal primary studies are needed to establish temporal precedence definitively. Second, most studies relied on self-report measures for all variables, which may inflate correlations due to common method bias. Although we corrected for measurement error using reliability coefficients, method variance may still affect our estimates. Third, the samples were predominantly from Western, educated, industrialized, rich, and democratic (WEIRD) contexts, limiting generalizability to other cultural settings.

#### Limitations of this meta-analysis

Our meta-analysis has several limitations that should be acknowledged. First, we were unable to pre-register our review protocol in PROSPERO or similar registries, as this study was initiated before the current emphasis on pre-registration in organizational psychology. Future meta-analytic studies from our research group will follow pre-registration procedures to enhance methodological transparency. Second, despite our comprehensive search strategy, we may have missed some unpublished studies, particularly dissertations and conference presentations. While our gray literature search and fail-safe N analyses suggest this is unlikely to substantially affect our conclusions, the possibility remains. Third, for some relationships (e.g., proactive behavior to career success), the number of available studies (*k* = 18) was relatively modest, limiting statistical power for subgroup and meta-regression analyses. Fourth, we examined proactive behavior as a composite construct due to limited primary studies examining specific proactive behaviors separately. Future meta-analyses with more primary studies could examine whether different types of proactive behavior (taking charge, voice, issue selling) have differential effects.

#### Future research directions

Our findings suggest several promising directions for future research. First, primary studies should employ longitudinal designs to establish causal relationships and examine how the proactive personality-career success relationship evolves over time. Second, research should examine boundary conditions–organizational factors such as climate for initiative, job autonomy, and supervisor support may moderate the effects of proactive personality and behavior on career outcomes. Third, future studies should examine objective career success indicators (salary, promotions) alongside subjective success to determine whether the mediating mechanisms differ across outcome types. Fourth, intervention studies are needed to test whether training programs can enhance proactive behavior and subsequently improve career outcomes. Finally, cross-cultural research should examine whether the relationships identified in this meta-analysis generalize to non-Western contexts, particularly collectivist cultures where proactive behavior may be perceived differently.

## Conclusion

This meta-analytic study provides a comprehensive examination of the mechanisms through which proactive personality influences subjective career success. The findings demonstrate that both job performance and proactive behavior serve as important mediators, with proactive behavior also influencing job performance in a sequential pathway. These findings contribute to the career success literature by elucidating the behavioral mechanisms underlying the proactive personality-career success relationship and provide practical guidance for organizations seeking to enhance employee career development. As organizations continue to navigate increasingly dynamic and uncertain environments, understanding and fostering proactive dispositions and behaviors will remain critical for both individual career success and organizational effectiveness.

## Data Availability

The original contributions presented in this study are included in the article/supplementary material, further inquiries can be directed to the corresponding author.
